# Enhancing the future of simulation-based education in pediatrics

**DOI:** 10.1186/s13052-021-00989-7

**Published:** 2021-02-17

**Authors:** Manuela Spadea, Massimiliano Ciantelli, Nicoletta Fossati, Armando Cuttano

**Affiliations:** 1 Centro di Formazione e Simulazione Neonatale NINA, U.O. Neonatologia, Dipartimento Materno-Infantile, Azienda Ospedaliero Universitaria Pisana, Pisa, Italy; 2 Department of Pediatric and Public Health Sciences, University of Turin, Turin, Italy; 3 U.O. Neonatologia, Dipartimento Materno-Infantile, Azienda Ospedaliero Universitaria Pisana, Via Roma, 67 Pisa, Italy; 4 St George’s University Hospitals, London, UK; 5 St George’s University of London, London, UK

**Keywords:** Simulation-based medical education, Pediatric education, Augmented reality systems, Serious games, Communication skills

## Abstract

Technology-enhanced simulation has emerged as a great educational tool for pediatric education. Indeed, it represents an effective method to instruct on technical and non-technical skills, employed by a large number of pediatric training programs. However, this unique pandemic era posed new challenges also on simulation-based education. Beyond the mere facing of the clinical and societal impacts, it is fundamental to take advantage from the current changes and investigate innovative approaches to improve the education of pediatric healthcare professionals. To this aim, we herein lay down the main pillars that should support the infrastructure of the future technology-enhanced simulation.

## Background

Simulation-based medical education (SBME) is a form of experiential learning that takes advantage of the use of simulation to create realistic clinical scenarios in a well-controlled environment [1]. By virtue of it, the trainee can improve their technical and non-technical skills through the interaction with other learners and the privilege of making mistakes safely, with corrective feedback from the simulator itself or their mentor [2]. Particularly in pediatrics, simulation has emerged as a powerful tool to enhance 360-degree medical education and has become part of pediatric residency and fellowship training programs [3, 4]. Furthermore, it may represent a method to boost skills even in other more experienced professionals (e.g. neonatologists) [5] or strengthen provider performance throughout real-time consultations with experienced clinicians while handling simulated resuscitation scenarios, using tele-medicine [6]. Nevertheless, considering at least procedural skills and teamwork behaviors, SBME usually requires strictly interactive and face-to-face educational sessions.

As senior instructors [7], we directly experienced that social distancing policy forced to suddenly stop simulation sessions, at least for groups of trainees. Therefore, during the COVID-19 period, SBME switched from shared simulation environments to e-learning platforms, virtual reality 360-degree videos [8] and individual *just-in-time* training (where a simulation center was located inside the hospital and the ward itself).

However, the efforts to avoid a gap in knowledge among professionals in this unique educational period could represent a great springboard for reshaping the future of SBME. Notwithstanding this unforeseen and impressive storm, we ought to take advantage of it. Indeed, if on the one hand we currently do not know how long this unique period will last, we are aware of the added value of simulation in pediatric education. Hence, we should strengthen what we still can achieve face-to-face, while enabling new perspectives for things that we will be forced to do remotely.

Indeed, as clinicians and researchers, we are witnessing a great expansion of diagnostic and prognostic tools, thanks to the introduction of newest technologies- e.g. Next Generation Sequencing (NGS) among the others- that are more and more available, even for the study of rare and complex pediatric pathologies, such as multiple sclerosis [9]. Moreover, as instructors, it is time for us to enable a technology enhanced environment in simulation-based learning. For this purpose, we herein provide some tips for reshaping SBME, striving to usher a new era in this essential element of pediatric education.

## Main text

### Multi-sensory augmentation as a key enabling factor for a new generation of pediatric simulation frameworks

New technologies should become part and parcel of procedural skills training. This could be the case of *Augmented Reality (AR) systems,* such as Microsoft HoloLens, Oculus devices or Google Glass*.* From this standpoint, their use could enable procedural skills teaching as if both mentor and trainee were in the same location, even if they work remotely. As an example, these devices could be used to capture the learner’s first-person view of a simulated emergency/delivery room through mixed reality capture, while the mentor’s hand gestures are captured through motion tracking (such as Leap Motion) and virtually displayed in the trainee’s AR space. These may represent valid solutions to exploit communication through visual channel.

However, vision is not the only sense one can use to provide information. Indeed, an alternative solution may be the use of tactile feedback. Touch is one of the most ancestral senses in Nature, which typically requires lower cognitive efforts as compared to audio/visual feedback. Because of this peculiarity, the use of tactile communication may introduce considerable benefits. Roboticists have proposed many solutions to convey a number of different stimuli, such as vibrations, normal and tangential forces, temperature. Many pilot studies investigated the use of this kind of stimuli in medical settings [10]. As an example, vibration-based feedback seems very effective in conveying directional cues, which may be used to naturally guide the arm of the trainee toward given targets. Force stimuli, e.g. through an engineered fabric band which squeezes the arm of the operator, could be used to codify “alert” signals. The very same technology, redesigned to fit at fingertip level, may be controlled to replicate pulsations, which for example could simulate the perception of arterial beats during palpation. Voice coils motors, finally, seem very effective in reproducing the contact with a surface, its roughness, and potentially its temperature with the addition of thermal displays. It would be fascinating to investigate this line of research, especially considering that haptic technologies may be integrated in existing AR/VR settings, opening highways of potential applications in SBME.

### Exploiting the available resources to improve non-technical skills

The learner could enhance their teamwork, leadership and clinical decision-making skills throughout the use of suitable *serious games* (games used with a pedagogical purpose) [11]*.* Meanwhile, the mentors could take advantage of the available recorded simulation sessions to assess what has been done before and find what can be improved, even with the help of experienced staff (e.g. counselors). This would result in a “meta-analysis” which could definitely lead to future tailor-made interventions in order to endlessly improve non-technical skill teaching.

Moreover, virtual e-learning platforms should be implemented with live webinars, which should be preferred to video-recorded lessons, to facilitate the debate among mentors and learners.

From this standpoint, debriefing sessions should be the core of e-learning education and video-assisted ones should be provided (even using previously video-recorded simulation sessions when a simulation session cannot be performed). This could enable the trainees to learn from (other people’s) mistakes.

### A focus on old and new problems, and keys on how to solve them

A great obstacle to these challenging perspectives could be funding resources. Indeed, the newest technologies often require conspicuous capital investments. Especially for rural hospitals, or low- and middle-income countries this could be unaffordable. For this reason, great effort should be put into devising and producing low-cost devices, to avoid the introduction of social disparities. Furthermore, it is worth mentioning that -at the current stage - the usage of such technologies does not require conspicuous investments. Indeed, their cost typically ranges between hundreds to a maximum of few thousand dollars, thanks to the extensive employment for other commercial purposes, such as electronic games.

On the other hand, governments and University institutions should guarantee their maximum effort in covering medical education costs, as this lays the foundation for a safer world while potentially sparing dreaded expensive medico-legal issues.

## Conclusions

In conclusion, we strongly believe that our mission is to guarantee high quality education, especially in the pediatric setting, notwithstanding the current pandemic. As described above, several opportunities are available to pursue this aim. Ultimately, we suggest keeping in mind an age-old truth: “As for the future, your task is not to foresee it, but to enable it”- Antoine de Saint-Exupéry, Le Petit Prince (1943).

## Data Availability

Not applicable.

## Abbreviation

SBME: Simulation-based medical education
